# Map the prevalence of irritable bowel syndrome across China: a systematic review and meta-analysis

**DOI:** 10.3389/fmed.2026.1740952

**Published:** 2026-03-25

**Authors:** Di Tian, Xinyu Liu, Huixia Qiao, Feng Liu

**Affiliations:** Department of Spleen and Stomach Diseases, Xi’an Hospital of Traditional Chinese Medicine, Xi'an, China

**Keywords:** China, irritable bowel syndrome, prevalence, systematic review, meta-analysis

## Abstract

**Background:**

Irritable bowel syndrome (IBS) is one of the most common functional gastrointestinal disorders worldwide. However, its prevalence varies substantially across countries and regions, reflecting heterogeneity in diagnostic approaches, study populations, and cultural factors. In China, which has the world’s largest population, the true burden of IBS remains uncertain. A comprehensive synthesis of prevalence estimates is needed to inform public health strategies and clinical care.

**Methods:**

We systematically searched Chinese and international databases from inception to August 2025 to identify observational studies reporting IBS prevalence in Chinese populations. Eligible studies were assessed for quality and extracted independently by two reviewers. Random-effects meta-analyses using logit transformation and random-intercept logistic regression models were applied. Subgroup analyses by sex, age group, and IBS subtype were performed. Heterogeneity was quantified using the *I*^2^ statistic and tau^2^.

**Results:**

A total of 65 studies (37 in Chinese and 28 in English) involving 197,764 participants and 23,341 IBS cases were included. Studies covered 23 provinces, with a concentration in Beijing, Shanghai, and Hubei, while no prevalence data were available for several regions including Hunan, Tianjin, Qinghai, and Xinjiang. The pooled prevalence of IBS in China was 11.0% (95% CI 9.0–13.4), with substantial heterogeneity (*I*^2^ = 99.4%). Sex-stratified analyses showed prevalence estimates of 9.0% (95% CI 6.8–11.8) in men and 11.1% (95% CI 8.1–14.9) in women, with no significant difference between sexes (*p* = 0.29). By age, prevalence was highest in adults aged 18–59 years (12.0, 95% CI 9.6–14.9), lower in the elderly (8.2, 95% CI 5.1–13.0), and rare among children (<18 years: 1.4, 95% CI 0.2–11.4). Subtype analysis showed comparable prevalence across IBS-C (3.9%), IBS-D (4.3%), IBS-M (3.0%), and IBS-U (2.7%).

**Conclusion:**

This systematic review and meta-analysis reveals that IBS affects approximately 1 in 10 Chinese adults, with considerable variation across regions and subgroups. The findings highlight the need for nationally representative studies, standardized diagnostic criteria, and targeted health policies to address the burden of IBS in China.

## Introduction

Irritable bowel syndrome (IBS) represents one of the most prevalent and clinically challenging functional gastrointestinal disorders worldwide, characterized by a complex interplay of abdominal pain, altered bowel habits, and bloating in the absence of structural pathology (1). Its global prevalence is estimated at approximately 3.8%–9.2%, though significant geographical variations exist, reflecting potential influences of genetic, environmental, dietary, and diagnostic factors (2–4). The socioeconomic burden of IBS is substantial, encompassing direct healthcare costs, reduced work productivity, and impaired quality of life that rivals inflammatory bowel disease in some populations (1, 4–6).

In China, one of the world’s most populous nation, understanding the epidemiology of IBS carries particular significance. The country has undergone unprecedented socioeconomic transformation, urbanization, and dietary shifts over recent decades, all factors hypothesized to influence IBS prevalence and manifestation (1–3, 7). However, the epidemiological landscape of IBS across China’s diverse regions remains inadequately characterized. Existing literature presents conflicting estimates, with reported prevalence rates ranging from under 3.66% to over 33.3%, suggesting substantial methodological heterogeneity or genuine regional variations (8, 9).

Several critical knowledge gaps persist in the Chinese IBS literature. First, the geographical distribution of research effort appears uneven, with potential over-representation of developed coastal regions and under-representation of inland provinces. Second, the impact of evolving diagnostic criteria (Rome I–IV) on prevalence estimates across the Chinese population has not been systematically evaluated. Third, while sex and age differences in IBS prevalence are established in Western populations, their patterns in the Chinese context require clarification. Finally, the distribution of IBS subtypes, constipation-predominant (IBS-C), diarrhea-predominant (IBS-D), mixed (IBS-M), and unsubtyped (IBS-U), across China’s population remains poorly documented.

This systematic review and meta-analysis aims to address these evidence gaps by comprehensively mapping IBS prevalence across China. Our specific objectives are to determine the pooled national prevalence of IBS, to analyse geographical variations and identify research-poor regions, to examine prevalence differences by sex, age, and diagnostic criteria, and to characterize the distribution of IBS subtypes. By providing a nuanced epidemiological portrait, this study seeks to inform clinical practice, guide resource allocation, and stimulate targeted research in underrepresented populations.

## Methods

### Search strategy and selection criteria

We conducted a systematic literature search following the Preferred Reporting Items for Systematic Reviews and Meta-Analyses (PRISMA) guidelines (10). Electronic databases including PubMed, Embase, Web of Science, Cochrane Library, China National Knowledge Infrastructure (CNKI), WanFang, and VIP Chinese Science and Technology Periodicals Database were searched from inception until August 2025. The search strategy combined keywords and Medical Subject Headings (MeSH) terms related to “irritable bowel syndrome,” “prevalence,” “epidemiology,” and “China” without language restrictions.

Studies were eligible for inclusion if they: (1) were original epidemiological investigations conducted in any region of China, including mainland China, Taiwan, Hong Kong, and Macao; (2) reported the prevalence of IBS based on recognized diagnostic criteria (Rome I, II, III, or IV); (3) included representative population samples (general population, students, specific occupational groups); and (4) provided sufficient data to calculate prevalence estimates and sample sizes. Reviews, case reports, and those focusing exclusively on clinical patients without a clear denominator were excluded.

### Data extraction and quality assessment

Two investigators independently screened titles and abstracts, followed by full-text assessment of potentially eligible articles. Any discrepancies were resolved through discussion or consultation with a third reviewer. A standardized data extraction form was used to collect the following information: first author, publication year, study period, geographical location (province), study design, sampling method, sample size, number of IBS cases, participant characteristics (age, sex, occupation), diagnostic criteria used (Rome I-IV), IBS subtype distribution (if available), and response rate. The methodological quality of included studies was assessed using the Joanna Briggs Institute (JBI) Critical Appraisal Checklist for Studies Reporting Prevalence Data by two investigators independently (11). The checklist comprises nine items evaluating sampling methods, sample size adequacy, study subject and setting description, data analysis coverage, validity of condition measurement, reliability of measurement, appropriateness of statistical analysis, and adequacy of response rate.

### Statistical analysis

All statistical analyses were performed using R software (version 4.3.0) with the meta and metafor packages. The primary outcome was the pooled prevalence of IBS with 95% confidence intervals (CIs). Given the anticipated substantial heterogeneity across studies, we employed a random-effects model using the DerSimonian-Laird method for all meta-analyses. Study-specific prevalence estimates were transformed using the logit transformation to stabilize variances before pooling, with back-transformation to the prevalence scale for presentation.

Between-study heterogeneity was quantified using the *I*^2^ statistic, with values of 25, 50, and 75% representing low, moderate, and high heterogeneity, respectively. The tau^2^ (*τ*^2^) statistic was used to estimate the between-study variance. Subgroup analyses were pre-specified to investigate potential sources of heterogeneity, including geographical region, sex, age groups (<18 years, 18–59 years, and ≥60 years), diagnostic criteria (Rome I–IV), IBS subtypes (IBS-C, IBS-D, IBS-M, and IBS-U), publication year, and study population characteristics (general population, students, and occupational groups). Differences between subgroups were tested using meta-regression for continuous variables (e.g., publication year) and subgroup analysis with tests for subgroup differences for categorical variables (e.g., diagnostic criteria).

Publication bias was assessed through visual inspection of funnel plots and statistical evaluation using Egger’s linear regression test and Begg’s rank correlation test. For proportion data analyzed using logit transformation, tests were conducted on the logit-transformed scale. Given the impact of high heterogeneity on funnel plot interpretation, we also performed contour-enhanced funnel plot analysis to distinguish between areas of statistical significance and potential publication bias patterns. All tests used a significance level of *α* = 0.05.

## Results

### Study characteristics

**Figure 1:**
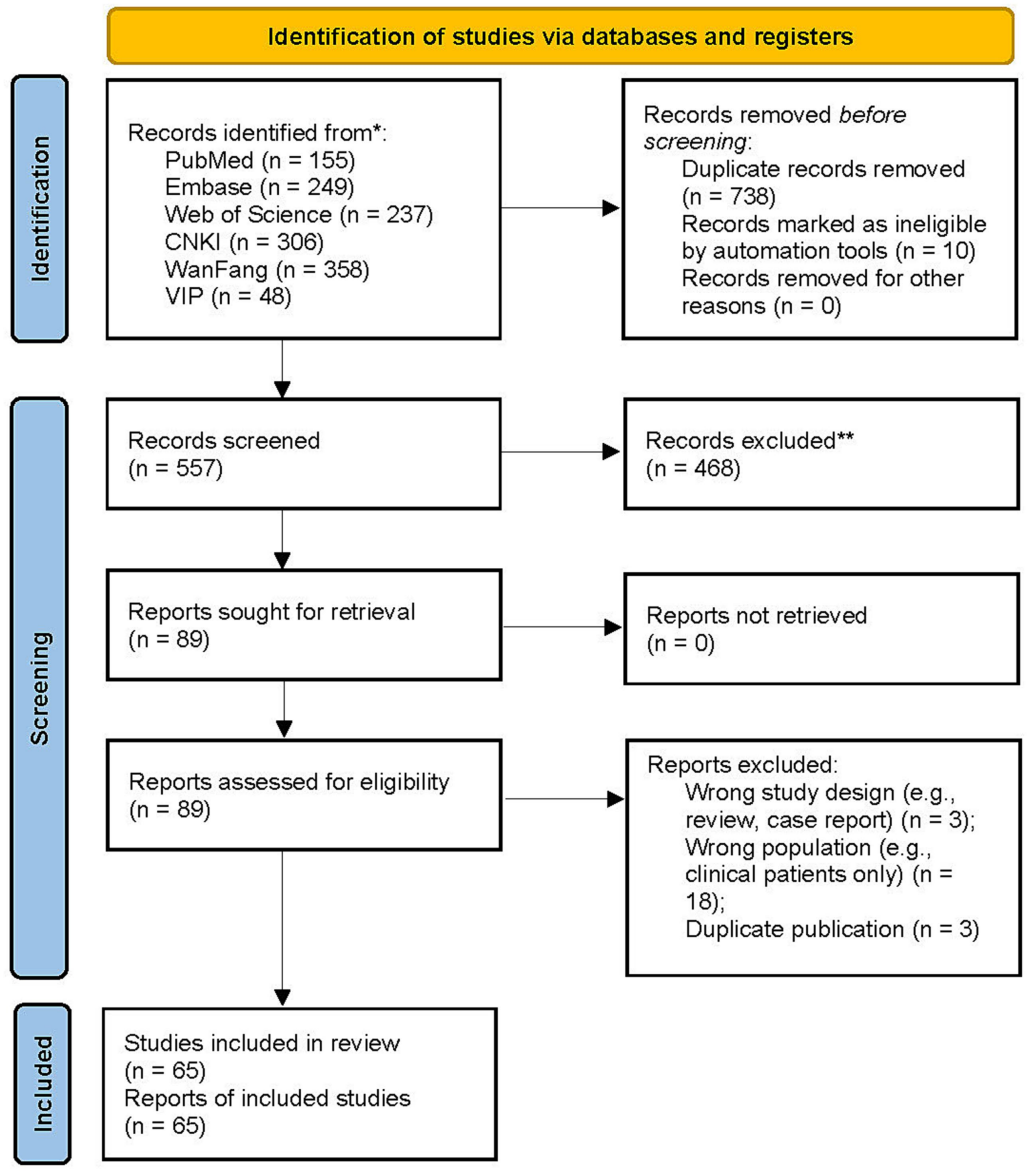
Study identification and selection flowchart. This PRISMA flow diagram illustrates the process of study identification, screening, eligibility assessment, and final inclusion in the systematic review and meta-analysis. The chart details the number of records identified through database searching, duplicates removed, records screened, full-text articles assessed for eligibility, and studies included in the quantitative synthesis, with specific reasons for exclusions at each stage.

Our systematic review and meta-analysis incorporated 65 independent studies, comprising a cumulative sample size of 197,764 participants, among whom 23,341 cases of irritable bowel syndrome (IBS) were identified. Overall, the methodological quality of the included studies was high. Most studies fulfilled all nine items of the JBI critical appraisal checklist, indicating appropriate sampling strategies, adequate sample sizes, valid and reliable measurement of outcomes, and suitable statistical analyses. All included studies met at least eight of the nine JBI criteria and were therefore considered to be of acceptable methodological quality. One study failed to meet the criterion relating to the validity of outcome measurement, but satisfied all remaining items. Based on the predefined appraisal criteria, all studies were retained for quantitative synthesis. Detailed results of the quality assessment are provided in Supplementary Table S1 (see Figure 1).

**Figure 2:**
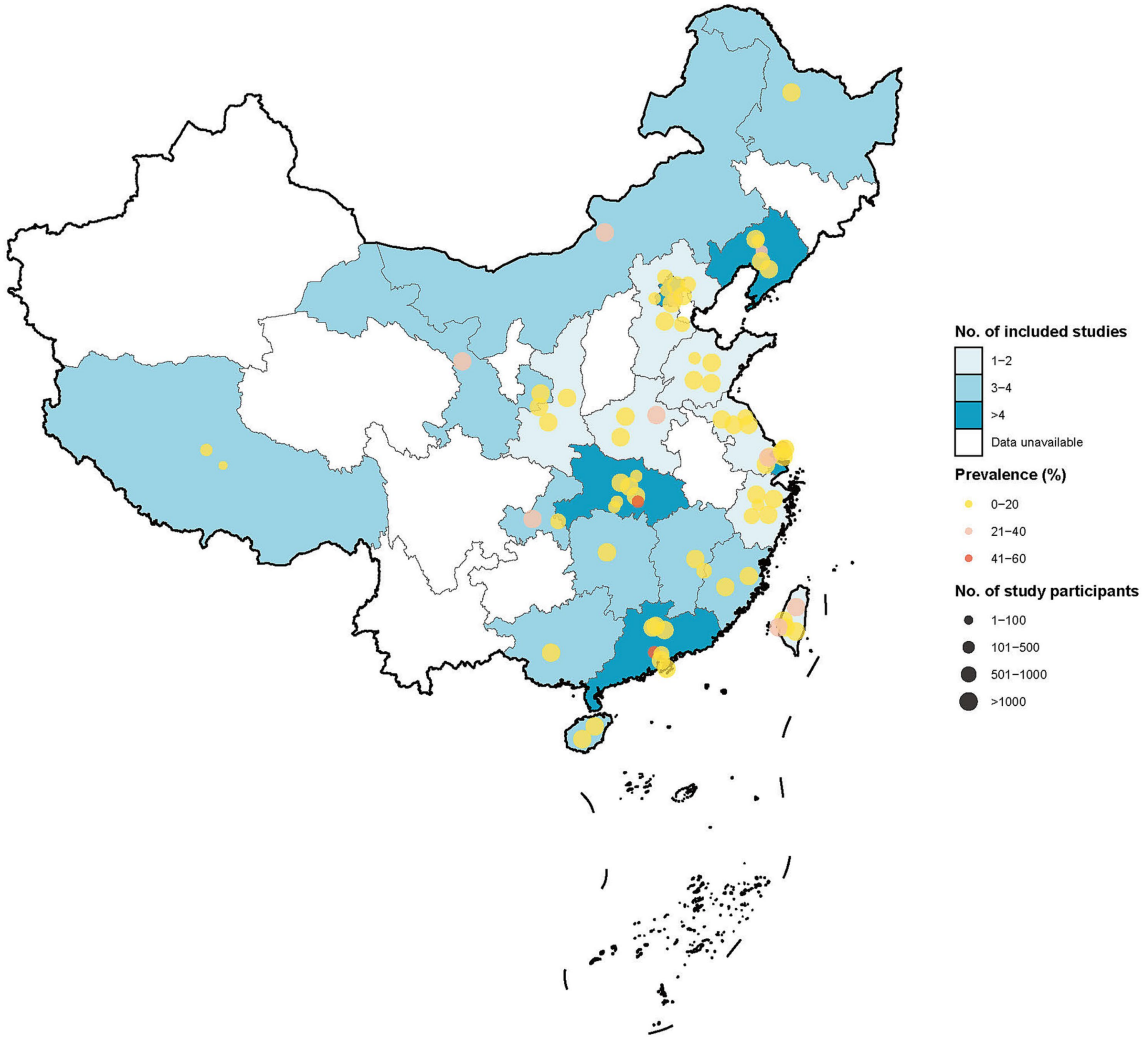
Geographical distribution of included studies and pooled prevalence of irritable bowel syndrome in China. Map of China showing the distribution and density of the 65 included studies across 23 provincial-level administrative regions. The color gradient indicates the number of studies conducted in each province, highlighting the concentration of evidence in Beijing, Shanghai, and Hubei, and the absence of data in several regions (e.g., Hunan, Tianjin, and Qinghai).

The literature base was linguistically diverse, with 37 studies published in Chinese and 28 in English. The geographical coverage of the included studies spanned 23 of China’s 34 provincial-level administrative regions. A notable unevenness was observed in the distribution of the evidence, with a high concentration of studies from major metropolitan areas such as Beijing, Shanghai, and Hubei province. Conversely, several regions, including Hunan, Tianjin, Qinghai, Xinjiang, and Guizhou, were identified as having no available prevalence data, highlighting significant geographical gaps in the literature. The sample sizes of the individual studies exhibited considerable variation, ranging from 312 to 16,500 participants. The number of reported IBS cases per study also varied substantially, with the majority of studies reporting fewer than 60 cases, though a subset reported larger case counts (see Figure 2).

### Overall prevalence

**Figure 3:**
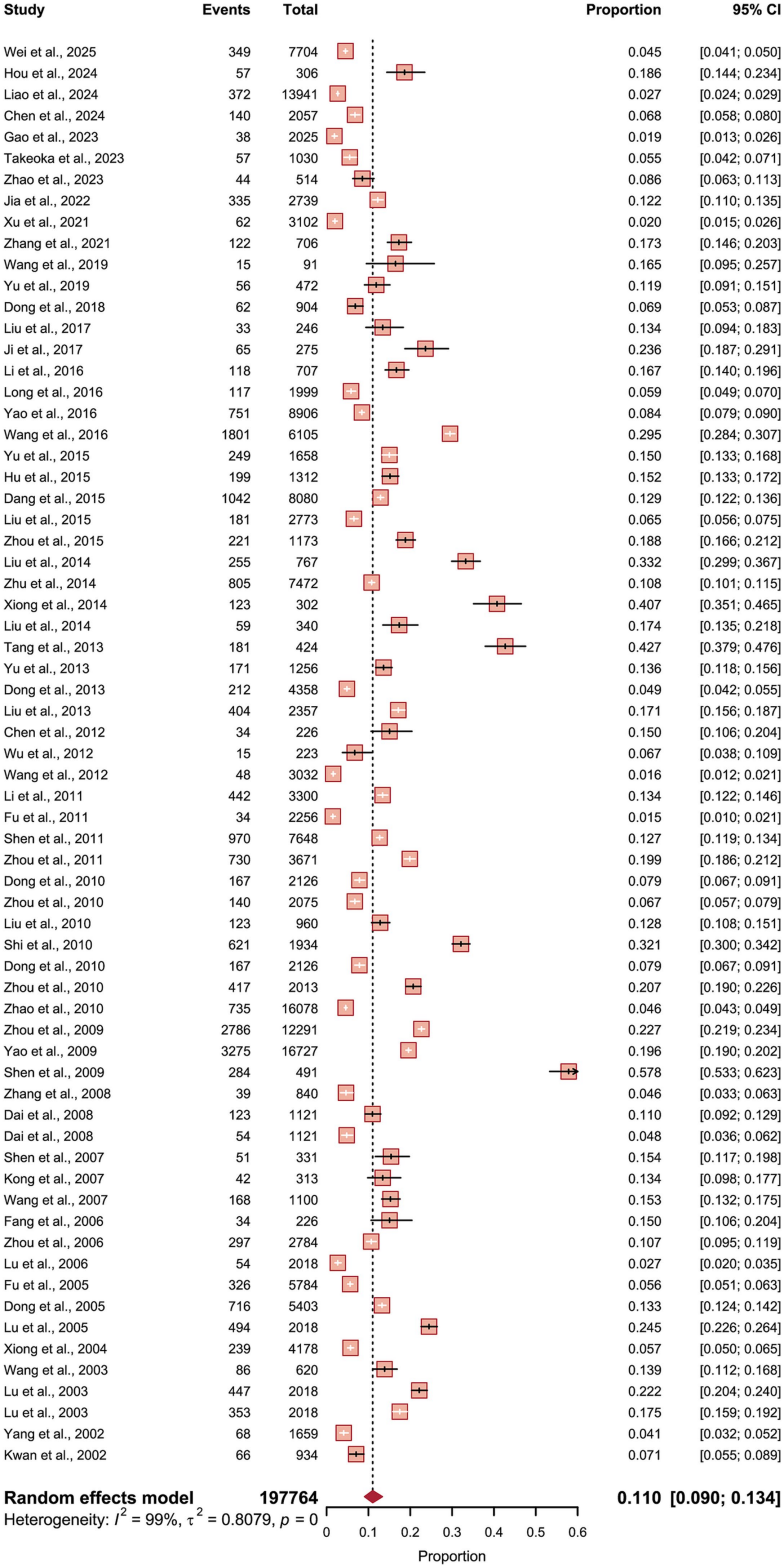
Pooled prevalence of irritable bowel syndrome in China. Forest plot of the overall pooled prevalence of IBS from the random-effects meta-analysis of 67 estimates. The summary estimate (diamond) and 95% confidence interval are shown at the bottom. The plot visually demonstrates the extreme heterogeneity (*I*^2^ = 99.4%) among individual study estimates (squares), with prevalence ranging from less than 2% to over 50%.

The random-effects meta-analysis, synthesizing data from 67 independent studies encompassing 197,764 individuals and 23,341 confirmed cases, determined the pooled prevalence of irritable bowel syndrome (IBS) across China to be 11.0% [95% confidence interval (CI) 9.0–13.4]. This synthesis was characterized by extreme and significant heterogeneity, as quantified by an *I*^2^ statistic of 99.4% (*τ*^2^ = 0.8079), indicating that the vast majority of the observed variance in prevalence estimates is attributable to genuine differences between studies rather than sampling error. The forest plot graphically confirmed this substantial variability, with individual study estimates spanning a remarkably wide range from less than 2% to over 57%. This broad dispersion in point estimates underscores the profound influence of differing study-level factors, such as geographic location, diagnostic criteria (Rome II, III, or IV), and specific population subgroups (e.g., students, soldiers, or general community dwellers), on the reported prevalence of IBS within the Chinese population (see Figure 3).

### Subtype prevalence

**Figure 4:**
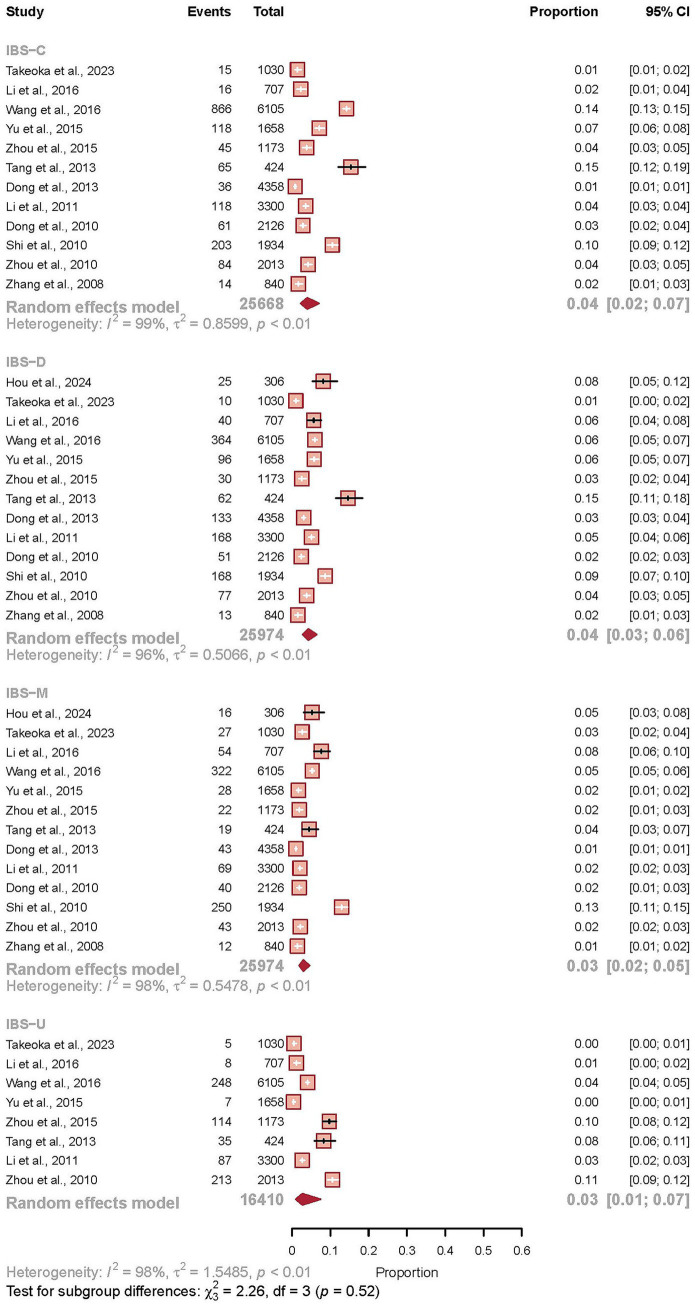
Pooled prevalence of irritable bowel syndrome subtypes in China. Forest plot displaying the pooled prevalence estimates for the four IBS subtypes based on a random-effects meta-analysis of 46 studies (*n* = 94,026). The subtypes are: irritable bowel syndrome with constipation (IBS-C), irritable bowel syndrome with diarrhea (IBS-D), mixed irritable bowel syndrome (IBS-M), and unsubtyped irritable bowel syndrome (IBS-U). For each subtype, the square represents the point estimate of prevalence and the horizontal line shows the 95% confidence interval. The size of each square is proportional to the statistical weight of the study in the meta-analysis. The diamond at the bottom of each subgroup represents the pooled prevalence and 95% confidence interval for that subtype. The test for subgroup differences indicated no statistically significant variation between subtypes (*p* = 0.52). Considerable heterogeneity was observed within each subtype (*I*^2^ > 95%).

The distribution of IBS subtypes was evaluated through a meta-analysis of 46 studies, encompassing 94,026 participants, of whom 4,540 were classified into a specific subtype. The analysis revealed broadly similar pooled prevalence estimates across the four principal subtypes. Irritable bowel syndrome with diarrhea (IBS-D) was the most frequently reported subtype with a prevalence of 4.3% (95% CI 2.8–6.5), closely followed by irritable bowel syndrome with constipation (IBS-C) at 3.9% (95% CI 2.2–7.0). The prevalence of mixed bowel habits (IBS-M) was 3.0% (95% CI 1.9–4.6), while the unsubtyped category (IBS-U) was the least common at 2.7% (95% CI 1.0–7.5). A test for subgroup differences confirmed that the variations between these subtype prevalences were not statistically significant (*p* = 0.52). Notably, the heterogeneity within each subtype remained substantial (*I*^2^ > 95%), indicating significant variation in subtype distribution between the individual studies, likely influenced by regional, diagnostic, or population-specific factors (see Figure 4).

### Influence of diagnostic criteria on prevalence estimates

The diagnostic criteria employed across studies demonstrated a notable impact on IBS prevalence estimates, though formal subgroup differences did not reach statistical significance (*p* = 0.069). Studies utilizing the Rome IV criteria (*k* = 8) yielded the lowest pooled prevalence at 5.5% (95% CI 2.7–11.0), approximately half the prevalence observed in studies using earlier criteria. In contrast, investigations employing Rome III criteria (*k* = 37) showed the highest prevalence at 12.5% (95% CI 9.7–15.9), while those using Rome II criteria (*k* = 20) demonstrated a similar estimate of 11.7% (95% CI 8.1–16.6). The limited data from studies using Rome I criteria (*k* = 2) showed a prevalence of 8.7% (95% CI 0.0–99.2), though this estimate was characterized by extreme uncertainty.

**Figure 5:**
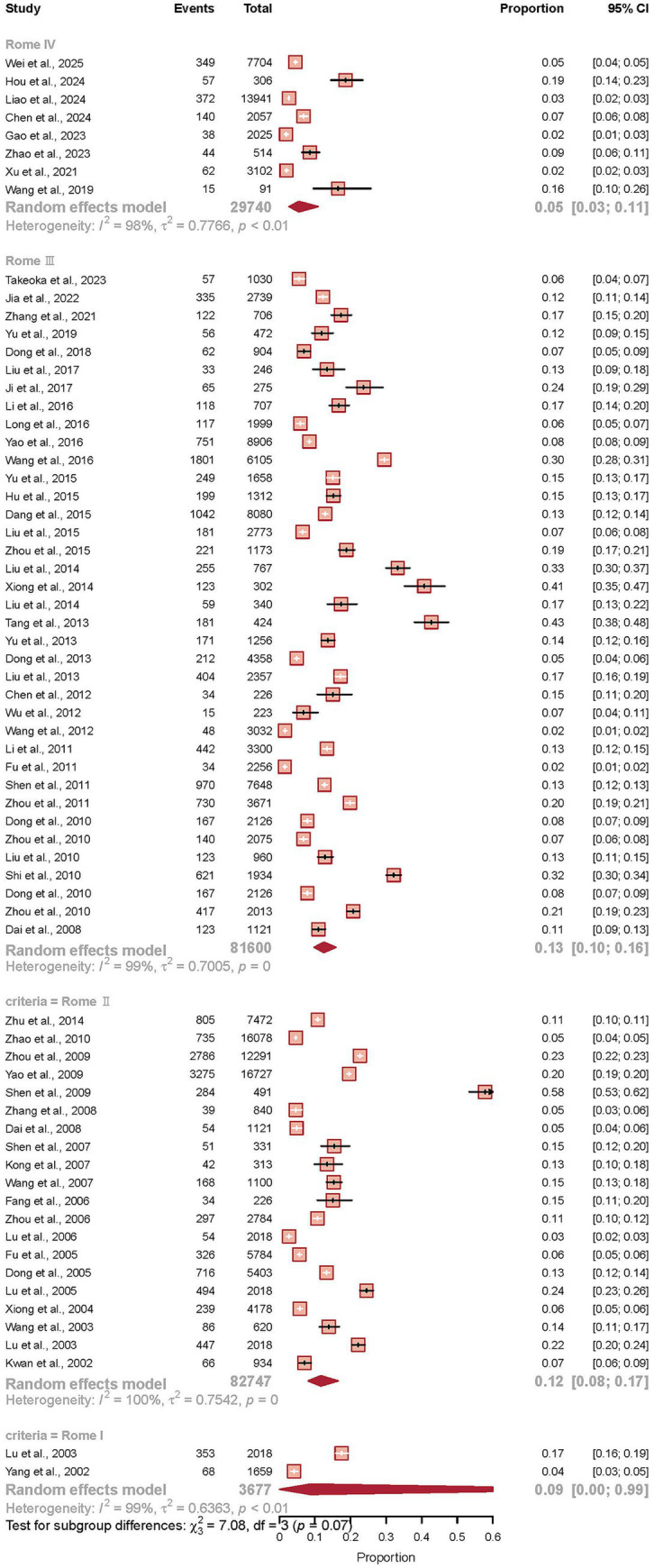
Influence of diagnostic criteria on irritable bowel syndrome prevalence in China. Forest plot presenting the pooled prevalence of IBS stratified by diagnostic criteria, based on a random-effects meta-analysis of 67 studies (*n* = 197,764). The analysis compared studies using Rome I, Rome II, Rome III, and Rome IV diagnostic criteria. Each study is represented by a square (point estimate) and horizontal line (95% confidence interval), with the square size proportional to the study’s statistical weight. The pooled estimates for each diagnostic criteria subgroup are represented by diamonds at the bottom of each subgroup. Prevalence estimates were 8.7% (95% CI 0.0–99.2) for Rome I, 11.7% (95% CI 8.1–16.6) for Rome II, 12.5% (95% CI 9.7–15.9) for Rome III, and 5.5% (95% CI 2.7–11.0) for Rome IV criteria. The test for subgroup differences indicated no statistically significant variation between diagnostic criteria (*p* = 0.069), though a clear gradient of decreasing prevalence was observed with newer diagnostic criteria. Substantial heterogeneity was observed within each subgroup (*I*^2^ > 98% for all criteria).

The chronological distribution of diagnostic criteria usage reveals an important methodological transition in the field. Rome II criteria predominated in earlier studies (2001–2010), Rome III criteria were most commonly employed during 2011–2019, while Rome IV criteria have been increasingly adopted in recent years (2020–2024). This temporal evolution in diagnostic standards partially confounds the assessment of genuine temporal trends in IBS prevalence, as the observed fluctuations may reflect both true epidemiological changes and methodological shifts. The lack of statistical significance in subgroup differences should be interpreted with caution, given the substantial numerical differences in point estimates and the limited number of studies using the most recent Rome IV criteria, which may constrain statistical power to detect true differences (see Figure 5).

### Sex-stratified prevalence

**Figure 6:**
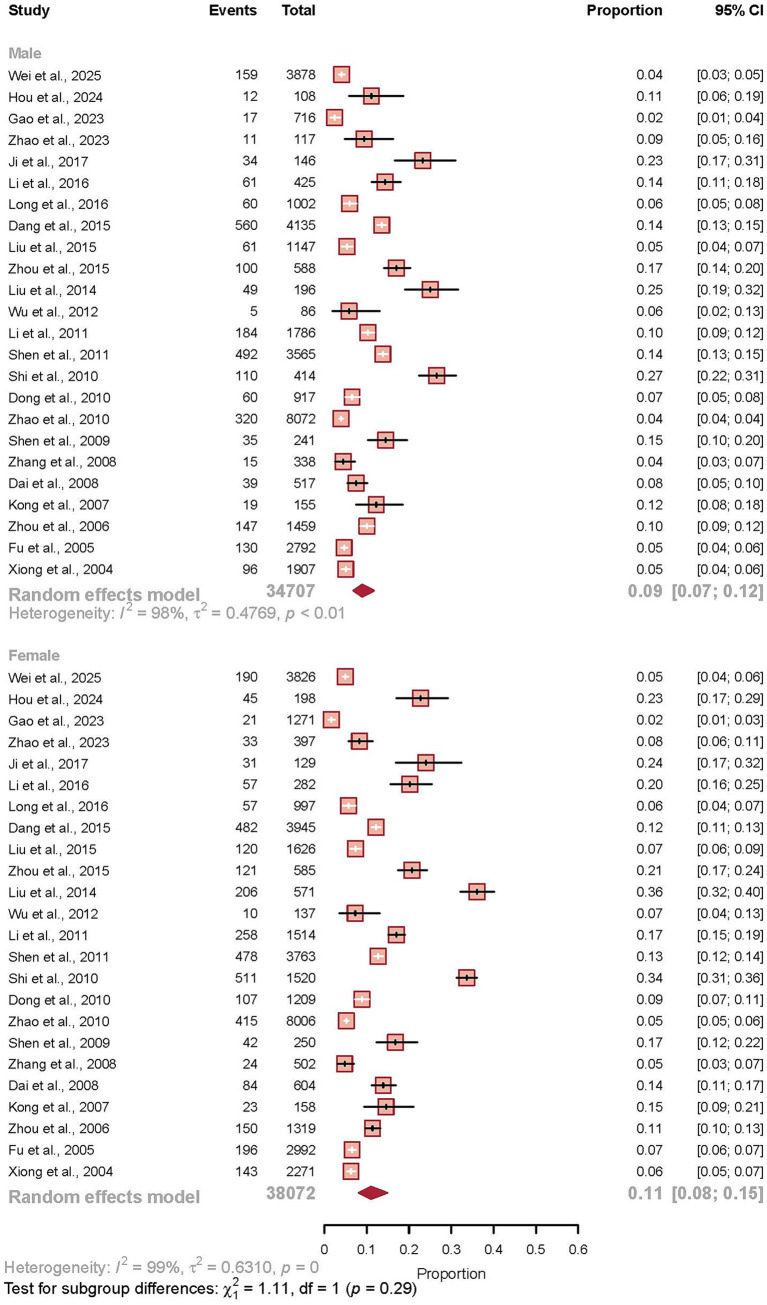
Sex-stratified prevalence of irritable bowel syndrome in China. Forest plot showing the pooled prevalence of IBS stratified by sex, derived from a random-effects meta-analysis of 48 studies (*n* = 72,779). The left panel displays results from 24 studies in male populations, while the right panel shows results from 24 studies in female populations. Each study is represented by a square (point estimate) and horizontal line (95% confidence interval), with the square size proportional to the study’s statistical weight. The pooled estimates for each sex are represented by diamonds at the bottom of each subgroup. The prevalence was 9.0% (95% CI 6.8–11.8) in men and 11.1% (95% CI 8.1–14.9) in women, with no statistically significant difference between groups (*p* = 0.29). Both subgroups exhibited substantial heterogeneity (*I*^2^ = 97.7% for men, *I*^2^ = 98.7% for women).

A subgroup analysis was conducted to evaluate sex-specific differences in IBS prevalence. Data from 48 studies, comprising 72,779 participants and 6,580 cases, were included in this analysis. The pooled prevalence was estimated at 9.0% (95% CI 6.8–11.8) for men and 11.1% (95% CI 8.1–14.9) for women. A formal test for subgroup differences indicated that this disparity was not statistically significant (*p* = 0.29). Despite the lack of statistical significance, the analysis revealed a consistent trend across most geographical regions, with female participants demonstrating a higher point prevalence of IBS in the vast majority of studied populations. Both subgroups exhibited considerable heterogeneity (*I*^2^ = 97.7% for men, *I*^2^ = 98.7% for women), suggesting that other unmeasured factors beyond sex contribute significantly to the variation in observed prevalence rates (see Figure 6).

### Age-stratified prevalence

**Figure 7:**
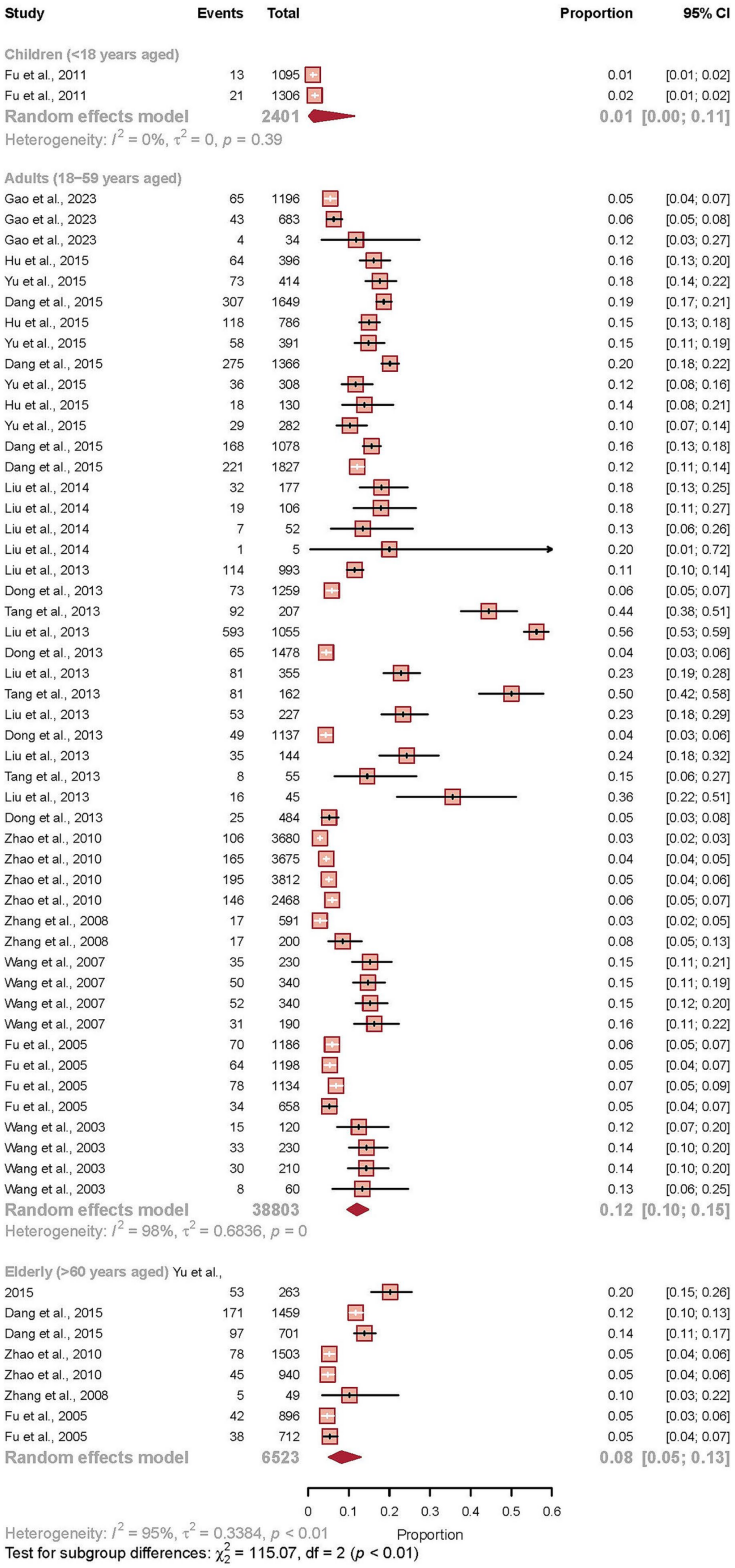
Age-stratified prevalence of irritable bowel syndrome in China. Forest plot presenting the pooled prevalence of IBS across different age groups based on a random-effects meta-analysis of 59 studies (*n* = 47,727). The analysis compared three age categories: children and adolescents (<18 years), adults (18–59 years), and elderly populations (≥60 years). Each study is represented by a square (point estimate) and horizontal line (95% confidence interval), with the square size proportional to the study’s statistical weight. The pooled estimates for each age group are shown as diamonds at the bottom of each subgroup. The test for subgroup differences revealed statistically significant variation between age groups (*p* < 0.001), with prevalence estimates of 1.4% (95% CI 0.2–11.4) in children, 12.0% (95% CI 9.6–14.9) in adults, and 8.2% (95% CI 5.1–13.0) in elderly populations. Substantial heterogeneity was observed within each age stratum (*I*^2^ > 95% for adults and elderly groups).

Analysis of 59 studies, comprising 47,727 participants with age-specific data, revealed significant disparities in IBS prevalence across different age groups (*p* for subgroup differences <0.001). The prevalence demonstrated a clear age-dependent pattern, with the lowest estimate observed in the pediatric population. Among children and adolescents under 18 years of age, the pooled prevalence was markedly low at 1.4% (95% CI 0.2–11.4). The prevalence increased substantially in early adulthood, peaking at 12.0% (95% CI 9.6–14.9) among adults aged 18 to 59 years, representing the group with the highest disease burden. In the elderly population aged 60 years and older, the prevalence subsequently declined to 8.2% (95% CI 5.1–13.0) (see Figure 7).

## Discussion

Our comprehensive meta-analysis of 67 studies encompassing nearly 200,000 participants reveals several key findings regarding the epidemiology of irritable bowel syndrome in China. The pooled prevalence of 11.0% establishes IBS as a common gastrointestinal disorder in the Chinese population, positioned within the mid-range of global estimates but substantially higher than the often-cited 4% global average (2). This discrepancy may reflect genuine population differences, methodological factors, or the unique socioeconomic transitions characterizing contemporary China (7, 9, 12).

The gradient of prevalence estimates across diagnostic criteria, from 12.5% (Rome III) to 5.5% (Rome IV), deserves particular attention. Although the test for subgroup differences did not reach statistical significance (*p* = 0.069), likely due to limited Rome IV studies, the halving of prevalence with newer criteria aligns with global observations of Rome IV’s stricter diagnostic thresholds (1–3). This pattern underscores the critical importance of standardized diagnostic approaches in future research and suggests that temporal comparisons of IBS prevalence must account for evolving diagnostic standards.

Epidemiological investigations into IBS reveal notable geographical heterogeneity, particularly when comparing Chinese populations within mainland China to those residing in Western nations, leading to what has been termed the “Chinese immigrant IBS paradox” (13). This phenomenon warrants comprehensive investigation, as existing direct comparative studies remain limited (14, 15). Current evidence suggests that the prevalence of IBS is higher among overseas Chinese populations, especially in Western, high-income countries, compared to their counterparts in mainland China (13, 16). For instance, global prevalence studies indicate significant variability in IBS rates, influenced by diagnostic criteria, survey methodologies, and cultural factors (3, 17–19).

This observed disparity is likely multifactorial, arising from complex gene–environment interactions that encompass dietary acculturation, altered microbiome exposures influenced by geography, psychosocial stressors related to acculturation and minority status, and systemic differences in healthcare access, diagnostic criteria application, and clinical awareness of functional gastrointestinal disorders (FGIDs) (13, 14, 16, 20). Functional gastrointestinal disorders (FGIDs), now often referred to as disorders of gut-brain interaction (DGBI), impact approximately 40% of the global population, with IBS being a prominent condition due to its complexity and impact on quality of life (17, 21, 22).

The remarkable stability of our pooled estimates across sensitivity analyses (leave-one-out range: 10.7–11.3%, Supplementary Figure S1) despite high heterogeneity suggests that methodological differences (including diagnostic criteria) may contribute substantially to between-study variation. This is particularly relevant for comparative studies across regions or time periods. The transition from Rome III to Rome IV criteria, with its approximately 50% reduction in prevalence estimates, provides a stark example of how diagnostic evolution alone can dramatically alter epidemiological understanding.

Our sex-stratified analysis revealed a consistent trend toward higher prevalence in women (11.1% vs. 9.0%), though this difference was not statistically significant (*p* = 0.29). This pattern contrasts with Western literature, which typically reports a significant female predominance with female-to-male ratios often exceeding 2:1 (4, 23). Several factors may explain this attenuated sex difference in the Chinese population (24, 25). First, cultural influences on symptom reporting and healthcare-seeking behavior may differ substantially from Western populations, potentially leading to under-reporting among women or increased medical consultation among men (1, 2, 26, 27). Second, methodological variations in study populations, particularly the overrepresentation of specific subgroups like military personnel and students in our included studies, may have obscured genuine sex differences present in the general population. Additionally, biological factors unique to Asian populations, including genetic predispositions and hormonal profiles, might contribute to a distinct epidemiological pattern (28–30). The substantial heterogeneity observed within both sex subgroups (*I*^2^ > 97%) further suggests that unmeasured regional and methodological factors are influencing these estimates. Future research should employ population-based sampling designs with careful attention to cultural and methodological factors that might affect sex-specific prevalence estimates in the Chinese context.

Our finding of peak IBS prevalence in the working-age population (18–59 years) carries substantial public health and economic implications. IBS in this demographic is associated with significant reductions in work productivity, increased absenteeism, and elevated healthcare utilization. Public health strategies should prioritize workplace wellness programs that include education on functional gastrointestinal disorders, stress management, and access to dietary counseling. For clinicians, a high index of suspicion for IBS in working-age adults presenting with chronic abdominal complaints is warranted, as early diagnosis and management can mitigate its socioeconomic impact.

The distribution of IBS subtypes showed IBS-D as the most common variant (4.3%), followed by IBS-C (3.9%), consistent with the results of Rome Foundation Global Study, different from the Western populations where IBS-C often predominates (3, 21). This pattern may reflect dietary factors, particularly the traditional Chinese diet’s composition, or genetic influences on bowel habit predisposition (31–33). The substantial heterogeneity within each subtype category indicates that additional factors, including regional dietary practices, microbiome variations, and diagnostic interpretation, likely influence subtype manifestation (30, 34–36). Future research should employ standardized subtyping and collect granular data on diet, microbiome, and genetic markers to elucidate the drivers of these geographical and clinical patterns.

Several limitations should be considered when interpreting our findings. The extreme heterogeneity, though explored through multiple subgroup analyses, suggests residual confounding by unmeasured study-level characteristics. Publication bias toward reporting higher prevalence rates is possible, though our funnel plot symmetry suggests this may be limited. The uneven geographical distribution of studies limits generalizability to underrepresented regions. Further, a key limitation of this meta-analysis is its reliance on study-level aggregated data, which precluded nuanced analyses of individual-level socioeconomic (e.g., income, education) and lifestyle (e.g., smoking, alcohol, detailed dietary habits) determinants. While some included studies reported associations with these factors, the metrics and categories were too heterogeneous to permit quantitative synthesis. Future primary studies should systematically collect and report standardized socioeconomic and lifestyle data to enable more informative pooled analyses and better inform targeted public health interventions. Finally, the meta-analytic approach inherently relies on study-level rather than individual-patient data, constraining more nuanced adjusted analyses.

Despite these limitations, our study provides the most comprehensive assessment to date of IBS epidemiology across China. The findings have several important implications. Clinically, the high prevalence underscores the need for increased awareness and evidence-based management strategies tailored to the Chinese population. From a public health perspective, the concentration of IBS in working-age adults highlights its potential economic impact through reduced productivity. For researchers, our identification of geographical and methodological gaps should guide future studies, particularly in underrepresented regions and using standardized Rome IV criteria.

## Conclusion

IBS is a common condition in China, affecting approximately 1 in 10 adults. Prevalence is highest among working-age adults, with women showing a modestly higher burden than men. The findings highlight the urgent need for nationally representative surveys, harmonized diagnostic criteria, and public health strategies to reduce disease burden. Establishing standardized epidemiological surveillance of IBS across China will be crucial for informing clinical management and health policy.

## Data Availability

Publicly available datasets were analyzed in this study. This data can be found at: https://zenodo.org/records/19198234.
