# Supplementary material for: Map the prevalence of irritable bowel syndrome across China: a systematic review and meta-analysis
Source: Front Med (Lausanne). 2026 Mar 25;13:1740952. doi: 10.3389/fmed.2026.1740952 (PMC13059203; doi:10.3389/fmed.2026.1740952)
Supplement: Supplementary file 1 [file Data_Sheet_1.docx]

### Supplementary Table S1. Methodological quality assessment of the studies included in the meta-analysis.

| Study | Item 1 | Item 2 | Item 3 | Item 4 | Item 5 | Item 6 | Item 7 | Item 8 | Item 9 | Overall appraisal |
| --- | --- | --- | --- | --- | --- | --- | --- | --- | --- | --- |
| Hou et al., 2024 | Yes | Yes | Yes | Yes | Yes | Yes | Yes | Yes | Yes | Include |
| Gao et al., 2023 | Yes | Yes | Yes | Yes | Yes | Yes | Yes | Yes | Yes | Include |
| Wang et al., 2019 | Yes | Yes | No | Yes | Yes | Yes | Yes | Yes | Yes | Include |
| Yu et al., 2019 | Yes | Yes | Yes | Yes | Yes | Yes | Yes | Yes | Yes | Include |
| Dong et al., 2018 | Yes | Yes | Yes | Yes | Yes | Yes | Yes | Yes | Yes | Include |
| Liu et al., 2017 | Yes | Yes | Yes | Yes | Yes | Yes | Yes | Yes | Yes | Include |
| Ji et al., 2017 | Yes | Yes | Yes | Yes | Yes | Yes | Yes | Yes | Yes | Include |
| Li et al., 2016 | Yes | Yes | Yes | Yes | Yes | Yes | Yes | Yes | Yes | Include |
| Yu et al., 2015 | Yes | Yes | Yes | Yes | Yes | Yes | Yes | Yes | Yes | Include |
| Hu et al., 2015 | Yes | Yes | Yes | Yes | Yes | Yes | Yes | Yes | Yes | Include |
| Dang et al., 2015 | Yes | Yes | Yes | Yes | Yes | Yes | Yes | Yes | Yes | Include |
| Liu et al., 2015 | Yes | Yes | Yes | Yes | Yes | Yes | Yes | Yes | Yes | Include |
| Zhou et al., 2015 | Yes | Yes | Yes | Yes | Yes | Yes | Yes | Yes | Yes | Include |
| Tang et al., 2013 | Yes | Yes | Yes | Yes | Yes | Yes | Yes | Yes | Yes | Include |
| Yu et al., 2013 | Yes | Yes | Yes | Yes | Yes | Yes | Yes | Yes | Yes | Include |
| Dong et al., 2013 | Yes | Yes | Yes | Yes | Yes | Yes | Yes | Yes | Yes | Include |
| Liu et al., 2013 | Yes | Yes | Yes | Yes | Yes | Yes | Yes | Yes | Yes | Include |
| Chen et al., 2012 | Yes | Yes | Yes | Yes | Yes | Yes | Yes | Yes | Yes | Include |
| Wu et al., 2012 | Yes | Yes | Yes | Yes | Yes | Yes | Yes | Yes | Yes | Include |
| Wang et al., 2012 | Yes | Yes | Yes | Yes | Yes | Yes | Yes | Yes | Yes | Include |
| Li et al., 2011 | Yes | Yes | Yes | Yes | Yes | Yes | Yes | Yes | Yes | Include |
| Fu et al., 2011 | Yes | Yes | Yes | Yes | Yes | Yes | Yes | Yes | Yes | Include |
| Shen et al., 2011 | Yes | Yes | Yes | Yes | Yes | Yes | Yes | Yes | Yes | Include |
| Dong et al., 2010 | Yes | Yes | Yes | Yes | Yes | Yes | Yes | Yes | Yes | Include |
| Zhou et al., 2010 | Yes | Yes | Yes | Yes | Yes | Yes | Yes | Yes | Yes | Include |
| Liu et al., 2010 | Yes | Yes | Yes | Yes | Yes | Yes | Yes | Yes | Yes | Include |
| Shi et al., 2010 | Yes | Yes | Yes | Yes | Yes | Yes | Yes | Yes | Yes | Include |
| Zhou et al., 2009 | Yes | Yes | Yes | Yes | Yes | Yes | Yes | Yes | Yes | Include |
| Yao et al., 2009 | Yes | Yes | Yes | Yes | Yes | Yes | Yes | Yes | Yes | Include |
| Zhang et al., 2008 | Yes | Yes | Yes | Yes | Yes | Yes | Yes | Yes | Yes | Include |
| Shen et al., 2007 | Yes | Yes | Yes | Yes | Yes | Yes | Yes | Yes | Yes | Include |
| Kong et al., 2007 | Yes | Yes | Yes | Yes | Yes | Yes | Yes | Yes | Yes | Include |
| Wang et al., 2007 | Yes | Yes | Yes | Yes | Yes | Yes | Yes | Yes | Yes | Include |
| Fang et al., 2006 | Yes | Yes | Yes | Yes | Yes | Yes | Yes | Yes | Yes | Include |
| Zhou et al., 2006 | Yes | Yes | Yes | Yes | Yes | Yes | Yes | Yes | Yes | Include |
| Fu et al., 2005 | Yes | Yes | Yes | Yes | Yes | Yes | Yes | Yes | Yes | Include |
| Wang et al., 2003 | Yes | Yes | Yes | Yes | Yes | Yes | Yes | Yes | Yes | Include |
| Liao et al., 2024 | Yes | Yes | Yes | Yes | Yes | Yes | Yes | Yes | Yes | Include |
| Long et al., 2016 | Yes | Yes | Yes | Yes | Yes | Yes | Yes | Yes | Yes | Include |
| Lu et al., 2006 | Yes | Yes | Yes | Yes | Yes | Yes | Yes | Yes | Yes | Include |
| Jia et al., 2022 | Yes | Yes | Yes | Yes | Yes | Yes | Yes | Yes | Yes | Include |
| Dong et al., 2010 | Yes | Yes | Yes | Yes | Yes | Yes | Yes | Yes | Yes | Include |
| Xu et al., 2021 | Yes | Yes | Yes | Yes | Yes | Yes | Yes | Yes | Yes | Include |
| Dong et al., 2005 | Yes | Yes | Yes | Yes | Yes | Yes | Yes | Yes | Yes | Include |
| Liu et al., 2014 | Yes | Yes | Yes | Yes | Yes | Yes | Yes | Yes | Yes | Include |
| Zhou et al., 2010 | Yes | Yes | Yes | Yes | Yes | Yes | Yes | Yes | Yes | Include |
| Yang et al., 2002 | Yes | Yes | Yes | Yes | Yes | Yes | Yes | Yes | Yes | Include |
| Takeoka et al., 2023 | Yes | Yes | Yes | Yes | Yes | Yes | Yes | Yes | Yes | Include |
| Zhu et al., 2014 | Yes | Yes | Yes | Yes | Yes | Yes | Yes | Yes | Yes | Include |
| Zhang et al., 2021 | Yes | Yes | Yes | Yes | Yes | Yes | Yes | Yes | Yes | Include |
| Chen et al., 2024 | Yes | Yes | Yes | Yes | Yes | Yes | Yes | Yes | Yes | Include |
| Lu et al., 2005 | Yes | Yes | Yes | Yes | Yes | Yes | Yes | Yes | Yes | Include |
| Shen et al., 2009 | Yes | Yes | Yes | Yes | Yes | Yes | Yes | Yes | Yes | Include |
| Zhou et al., 2011 | Yes | Yes | Yes | Yes | Yes | Yes | Yes | Yes | Yes | Include |
| Xiong et al., 2014 | Yes | Yes | Yes | Yes | Yes | Yes | Yes | Yes | Yes | Include |
| Zhao et al., 2023 | Yes | Yes | Yes | Yes | Yes | Yes | Yes | Yes | Yes | Include |
| Zhao et al., 2010 | Yes | Yes | Yes | Yes | Yes | Yes | Yes | Yes | Yes | Include |
| Kwan et al., 2002 | Yes | Yes | Yes | Yes | Yes | Yes | Yes | Yes | Yes | Include |
| Yao et al., 2016 | Yes | Yes | Yes | Yes | Yes | Yes | Yes | Yes | Yes | Include |
| Liu et al., 2014 | Yes | Yes | Yes | Yes | Yes | Yes | Yes | Yes | Yes | Include |
| Wang et al., 2016 | Yes | Yes | Yes | Yes | Yes | Yes | Yes | Yes | Yes | Include |
| Lu et al., 2003 | Yes | Yes | Yes | Yes | Yes | Yes | Yes | Yes | Yes | Include |
| Wei et al., 2025 | Yes | Yes | Yes | Yes | Yes | Yes | Yes | Yes | Yes | Include |
| Dai et al., 2008 | Yes | Yes | Yes | Yes | Yes | Yes | Yes | Yes | Yes | Include |
| Xiong et al., 2004 | Yes | Yes | Yes | Yes | Yes | Yes | Yes | Yes | Yes | Include |

Quality assessment of the studies included in the meta-analysis were conducted using the Joanna Briggs Institute (JBI) Critical Appraisal Checklist for Studies Reporting Prevalence Data. The checklist comprises nine items evaluating sampling methodology, sample size adequacy, description of study subjects and setting, data analysis coverage, validity and reliability of outcome measurement, appropriateness of statistical analysis, and adequacy of response rate. Each study was independently assessed by two reviewers, with each item rated as “Yes”, “No”, “Unclear”, or “Not applicable”. Overall appraisal reflects whether studies were considered suitable for inclusion in the quantitative synthesis.


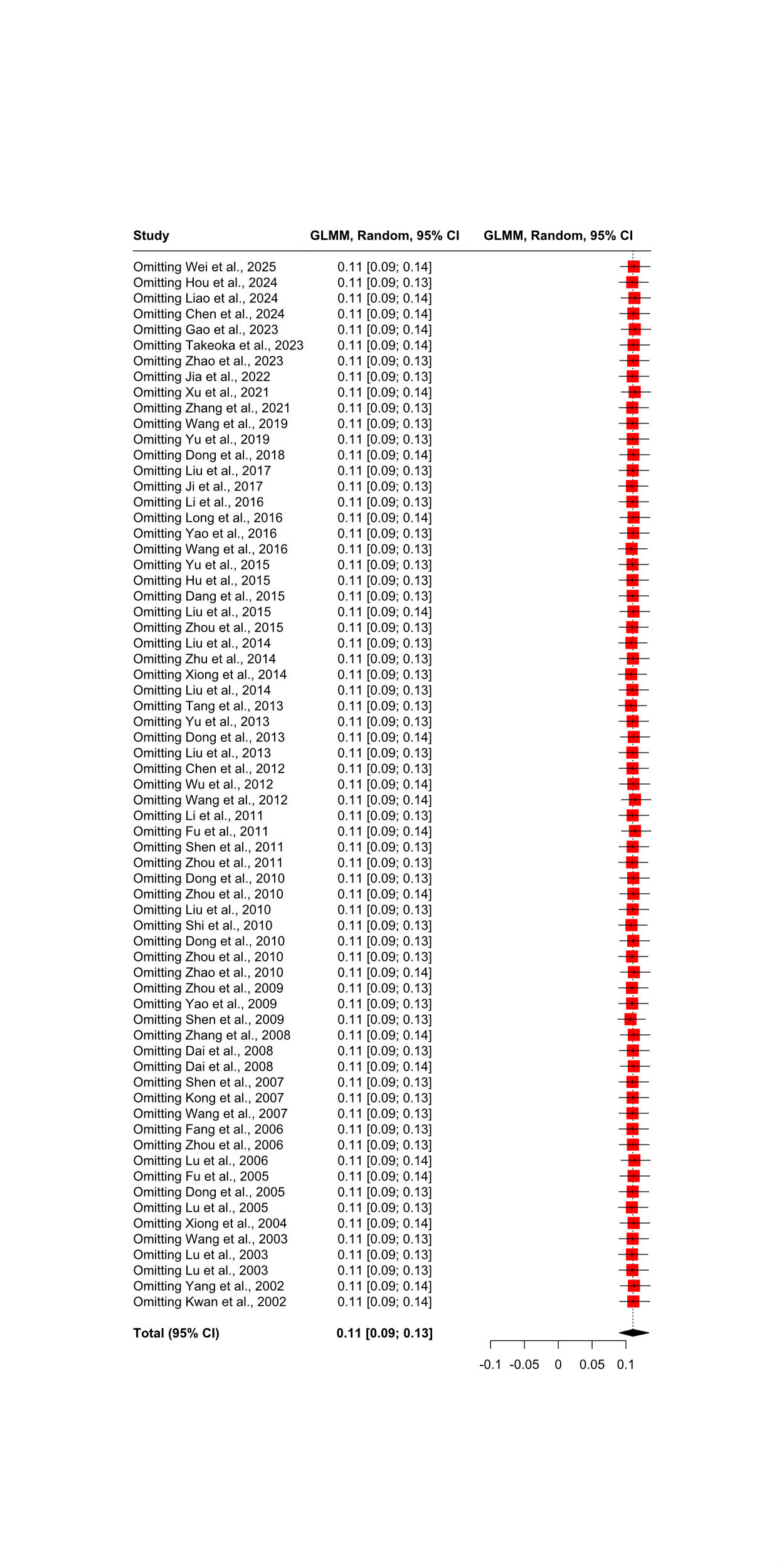


### Supplementary Figure S1. Leave-one-out sensitivity analysis of irritable bowel syndrome prevalence in China using random-effects model.


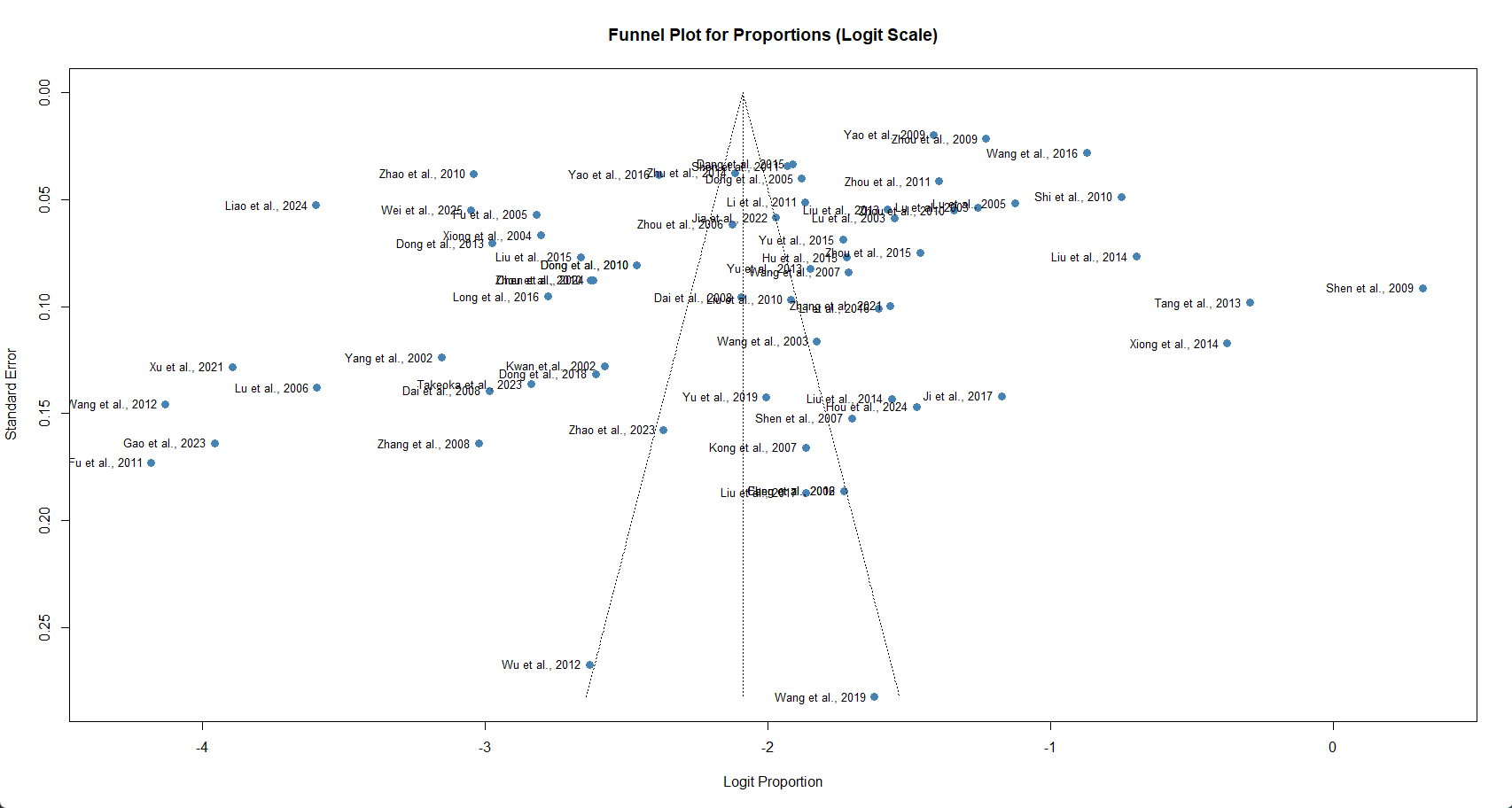


### Supplementary Figure S2. Funnel plot for publication bias assessment.

The p-value for Egger’s linear regression test and Begg’s rank correlation test were 0.116 and 0.372, respectively.
